# Automatic Scoring of Semantic Fluency

**DOI:** 10.3389/fpsyg.2019.01020

**Published:** 2019-05-16

**Authors:** Najoung Kim, Jung-Ho Kim, Maria K. Wolters, Sarah E. MacPherson, Jong C. Park

**Affiliations:** ^1^School of Computing, Korea Advanced Institute of Science and Technology, Daejeon, South Korea; ^2^School of Informatics, University of Edinburgh, Edinburgh, United Kingdom; ^3^Human Cognitive Neuroscience, Department of Psychology, University of Edinburgh, Edinburgh, United Kingdom

**Keywords:** verbal fluency, semantic fluency, executive function, semantic memory, word embeddings, relation extraction, category fluency test

## Abstract

In neuropsychological assessment, semantic fluency is a widely accepted measure of executive function and access to semantic memory. While fluency scores are typically reported as the number of unique words produced, several alternative manual scoring methods have been proposed that provide additional insights into performance, such as clusters of semantically related items. Many automatic scoring methods yield metrics that are difficult to relate to the theories behind manual scoring methods, and most require manually-curated linguistic ontologies or large corpus infrastructure. In this paper, we propose a novel automatic scoring method based on Wikipedia, Backlink-VSM, which is easily adaptable to any of the 61 languages with more than 100k Wikipedia entries, can account for cultural differences in semantic relatedness, and covers a wide range of item categories. Our Backlink-VSM method combines relational knowledge as represented by links between Wikipedia entries (*Backlink model*) with a semantic proximity metric derived from distributional representations (*vector space model*; VSM). Backlink-VSM yields measures that approximate manual clustering and switching analyses, providing a straightforward link to the substantial literature that uses these metrics. We illustrate our approach with examples from two languages (English and Korean), and two commonly used categories of items (animals and fruits). For both Korean and English, we show that the measures generated by our automatic scoring procedure correlate well with manual annotations. We also successfully replicate findings that older adults produce significantly fewer switches compared to younger adults. Furthermore, our automatic scoring procedure outperforms the manual scoring method and a WordNet-based model in separating younger and older participants measured by binary classification accuracy for both English and Korean datasets. Our method also generalizes to a different category (fruit), demonstrating its adaptability.

## 1. Introduction

The semantic (or category) fluency task consists of verbally naming as many words from a single category as possible in sixty seconds. Performance on this task is sensitive to variation in executive function and semantic memory (Tombaugh et al., 1999; Mathuranath et al., 2003; Henry et al., 2004; Mioshi et al., 2006; McDowd et al., 2011; Maseda et al., 2014). Semantic fluency performance can successfully differentiate between people with (mild) Alzheimer's Disease (Locascio et al., 1995; Salmon et al., 2002) and healthy older controls. Hence, it is often included as a subtest within larger cognitive test batteries that aim to discover signs of cognitive decline (Dubois et al., 2000; Mioshi et al., 2006; Weintraub et al., 2009; König et al., 2018).

Typically, semantic fluency is scored by counting the number of correct unique semantic category items produced (Lezak, 2004; Hazin et al., 2016). While the total count score provides clinicians a quick and easy measure, more detailed qualitative analysis for scoring semantic fluency data can provide additional insights into human cognitive performance (Troyer et al., 1997; Mayr and Kliegl, 2000; Troyer, 2000; Abwender et al., 2001).

However, existing manual scoring procedures for these detailed analyses are considered time-consuming as they require careful reanalysis of the sequence of words produced. In addition, manual scoring procedures that have been developed for one language, cultural background, and semantic category may need to be substantially altered and revalidated in different contexts. For example, the Chinese Zodiac is well-known in Korean folk culture and East Asian culture in general, and the twelve animal gods (e.g., *rat, ox, tiger, rabbit*, etc.) form a meaningful semantic cluster that Korean participants often exploit to produce animal name sequences.

To address these issues, in the past decade, researchers have proposed a number of different automatic scoring methods (Prescott et al., 2006; Sumiyoshi et al., 2009; Pakhomov et al., 2012, 2016; Clark et al., 2014, 2016; Nicodemus et al., 2014; Voorspoels et al., 2014; Linz et al., 2017b; König et al., 2018). Since most of these approaches propose sets of novel metrics, it can be difficult to relate their scores to a large body of literature reporting scores from established manual scoring methods. Moreover, as some of these methods require carefully curated lexical resources, they can be difficult to port to new languages and new semantic categories (such as supermarket items or fruits) that tend to be underrepresented in traditional lexical databases.

In this paper, we propose and evaluate a Wikipedia-based method—the Backlink-Vector Space Model (Backlink-VSM)—that combines semantic proximity metrics with the extensive knowledge about relations between concepts that is encoded in links between Wikipedia entries. The Vector Space Model (VSM) was previously designed as a part of the automatic category fluency data analysis described in Wolters et al. (2016) and has also been used in Linz et al. (2017a,b), König et al. (2018), and Paula et al. (2018). The brief outline of VSM given in Wolters et al. (2016) is elaborated, and the limitations of standalone VSM are reinforced by introducing the Backlink model. We only focus on the lexical analysis of category fluency; the acoustic/prosodic analysis, as discussed in Wolters et al. (2016) and König et al. (2018), is not within the scope of this paper.

Our method produces results that correlate well with the manual clustering and switching metrics introduced by Troyer and colleagues (Troyer et al., 1997; Troyer, 2000) and can replicate known differences in clustering and switching patterns between younger and older people. Given that most of our work is based on open-source software and publicly available data, our results can be easily adapted and reproduced by other researchers for different languages.

The paper is structured as follows. In section 2, we provide a brief overview of existing manual and automatic scoring methods. The Backlink-VSM method is described in detail in section 3. Evaluation and discussion of the results for English and Korean data are documented in sections 4 and 5, and plans for further extending and evaluating the method are outlined in section 6.

## 2. Background

### 2.1. Manual Scoring Methods Beyond Word Counts

Semantic fluency performance has traditionally been reported, and continues to be measured, mostly using the total number of correct words produced by a participant within a given semantic category (e.g., animals). To complement word counts, Troyer and colleagues (Troyer et al., 1997) suggested using *clustering* and *switching* as finer probes of cognitive dysfunction. Clustering and switching are based on the observation that participants performing semantic fluency tend to produce word chains that are grouped into semantic subcategories (*clusters*), and changes from one subcategory to another, which are called *switches* (Abwender et al., 2001). This type of analysis has been used extensively in the literature (Tröster et al., 1998; Koren et al., 2005; Murphy et al., 2006; Haugrud et al., 2010).

Clustering and switching metrics have been shown to distinguish Alzheimer's Disease and Parkinson's Disease patient groups from their respective control groups, whereas mere word counts do not (Troyer et al., 1998). The normative data presented in Troyer (2000) demonstrate that these measures are also sensitive to cognitive decline caused by healthy aging, differentiating between younger and older healthy adult groups. Specifically, older adults produce less switches than younger adults, but the average length of the produced clusters is comparatively unaffected by age.

A detailed annotation protocol for determining clusters and switches in sequences of animal names has been described in Troyer et al. (1997). The protocol lists a fixed set of possible clusters based on various categories, such as taxonomy or living environment (e.g., *Living Environment–Africa: rhinoceros, tiger, zebra*…*/Australia: emu, kangaroo, kiwi*…*, Zoological Categories–Bird: budgie, condor, eagle*…*/Feline: bobcat, cat, cheetah* … etc.).

While this protocol has been validated and shown to have good inter-rater reliability for American English, extending it to different languages and cultures is not straightforward. New categories that are relevant for the particular culture (e.g., Chinese Zodiac animals) need to be introduced and defined, and existing clusters must be altered or augmented taking cultural, regional and linguistic factors into consideration. Depending on the extent of the changes, the resulting protocol may then need to be validated again.

Several alternative protocols for determining clusters and subgroups have been proposed. For instance, Abwender et al. (2001) suggested decomposing switching into subtypes depending on whether a transition occurs between clustered items or non-clustered items (single words), claiming these different subtypes of switching could tap into different cognitive processes. March and Pattison (2006) have reported that subcategory counts were more informative than the original clustering and switching metrics in distinguishing individuals with Alzheimer's Disease from older controls.

Not all fine-grained analyses of semantic fluency data rely on notions of subgroups. Rohrer et al. (1995) developed a framework that focuses on the time required to access the *i*th word *w*_*i*_ in a sequence of *n* words. The response time *t*_*i*_ is defined as the time between the end of the word *w*_*i*−1_ and the start of the word *w*_*i*_, including any hesitations, verbal comments, and other vocalizations, such as laughter. The response times of each speaker are used to create a linear model (Rohrer et al., 1995; Mayr and Kliegl, 2000) given in Equation 1.

(1) $$\begin{array}{l} t_{n}=c+s*n \end{array}$$

In this model, *c* is a lexical retrieval constant and *s* models gradual increases in retrieval time. McDowd et al. (2011) have shown that these item response times are sensitive to cognitive aging and cognitive impairment.

### 2.2. Automatic Analysis Methods

Manual analyses have several drawbacks:

- They are time-consuming to adapt to new languages, cultures, and task domains (e.g., supermarket items, kitchen utensils) as this requires a revision and potential revalidation of the annotation protocols.
- They are time-consuming to conduct and require well-trained annotators.
- Making decisions about words and clusters that are not included in existing annotation manuals can introduce inconsistencies and inaccuracies, especially when clinicians and researchers are working on their own.

Automatic analysis methods can address most of these issues:

- If given a large enough dataset for a new language, culture, and task domain, one is only required to retrain the statistical models that underpin the automatic analysis algorithm.
- Human annotators do not need to be trained; once the statistical models have been constructed, analysis is almost immediate, given a transcribed string of words.
- Words that are not covered by existing annotation manuals are dealt with in a consistent way, while the clusters that can be detected are only limited by the size and content of the underlying training materials.

There are three main approaches to the automatic analysis of semantic fluency data. In the first approach, novel semantic similarity metrics between two adjacent words are derived from corpora of documents, curated databases, or data sets of semantic fluency task responses and other word association tasks. These are then used to characterize a participant's performance on a semantic fluency task. For example, Pakhomov et al. (2012) present a series of measures that are based on WordNet, a large, manually curated taxonomy of words and concepts, and that can differentiate between healthy aging and cognitive impairment. Latent Semantic Analysis has been used to extract relevant word similarity metrics from documents (Pakhomov and Hemmy, 2013; Nicodemus et al., 2014), and Pakhomov et al. (2016) use statistics of word co-occurrence derived from semantic fluency task responses and word association tests.

In the second approach, semantic fluency data are used to construct models of the semantic memory of different populations (e.g., schizophrenia/healthy control or dementia/healthy control). These studies use data reduction and clustering to aggregate data on commonly co-occurring words in both category fluency data and other linguistic data (Chan et al., 1993; Prescott et al., 2006; Sumiyoshi et al., 2009; Voorspoels et al., 2014).

The third approach attempts to replicate the results of manual analyses using an algorithm based on automatically computed word similarity measures. In contrast to the first approach, which essentially proposes a new set of metrics, a replication of existing manual schemes makes it easier to relate findings from automated analyses to the substantial literature that uses manual classifications. A basic automation of an existing scoring procedure has been utilized by Haugrud et al. (2011), Clark et al. (2014), and Clark et al. (2016), aiming at an exact replication of the established protocol to compute the results more efficiently and with better consistency. Linz et al. (2017b) use word similarity measures derived from a very large corpus of web pages (Baroni et al., 2009).

In this paper, we propose a novel solution that combines information about the relationship between different concepts (through the Backlink model) with information about the semantic similarity of two words (through the vector space model), while maintaining a connection to the literature by using clustering and switching patterns as the main evaluation metrics.

## 3. The Backlink-VSM Method

The Backlink-VSM method combines two different methods for determining cluster boundaries that are designed to complement each other qualitatively. The first method (*Vector Space* model; VSM, c.f. section 3.1) models similarities between words in a vector space using word embeddings. A preliminary version of this model was described in Wolters et al. (2016) and evaluated on a small Korean dataset of 20 speakers; the model we present here is revised. The second method (*Backlink* model, c.f. section 3.2) augments the vector space model with relational information derived from link structures in Wikipedia. The Backlink model is capable of capturing document-level information that the VSM cannot, and the VSM provides a systematic way of resolving out-of-vocabulary and compound expressions that the Backlink model does not.

### 3.1. Vector Space Model

The vector space model is based on a distributional model of word meaning, where the meaning of a word is characterized by the words with which it co-occurs. Such models are commonly used in computational linguistics and computational psycholinguistics, and may reveal structures within the mental lexicon that cannot be captured through taxonomic or categorical relationships (Hills et al., 2012).

The idea of using co-occurrence patterns for automatic analysis of category fluency is not novel (Chan et al., 1993; Prescott et al., 2006; Sung et al., 2012). However, Voorspoels et al. (2014) argue that analyses based on simple co-occurrence patterns are not reliable, since semantically related words are not necessarily directly adjacent^1^.

In our model, we use vector representations of words (*word embeddings*) that can be calculated from their distribution in a large corpus to model their meanings. A good word embedding model is expected to generate vectors that are close to each other for words that are similar in meaning.

Within this *vector space* of word meanings, we can easily calculate the proximity of word vectors using cosine similarity (Equation 2; −1 ≤ cos θ ≤ 1), with 0 representing orthogonality (no relation) and values close to 1 representing high similarity.

(2) $$\begin{array}{l} \cos\theta =\frac{\vec{a}\cdot \vec{b}}{∥\vec{a}∥\;∥\vec{b}∥} \end{array}$$

A well-known method for mapping words to vectors is Latent Semantic Analysis (LSA) (Landauer et al., 1998). Although this method typically uses word-document matrices, it has been previously applied to the assessment of semantic fluency data in relation to schizophrenia using word co-occurrence matrices (Nicodemus et al., 2014). However, word embeddings learned by neural networks based on context prediction significantly outperform other approaches, including LSA, in tasks, such as measuring semantic relatedness and detecting syntactic regularities (Mikolov et al., 2013c; Baroni et al., 2014; Linz et al., 2017a; Paula et al., 2018). For this reason, we adopt the latter approach for our automatic analysis. The particular architecture we use is the word2vec model (Mikolov et al., 2013b), that estimates the vector representation of a word by a neural network that predicts its context given the words that surround the word of interest. The computation becomes more expensive as the number of surrounding words that are considered (i.e., the *context window*) increases. The context window is usually not large, with 2-10 words on either side being the common choice in practice (Mikolov et al., 2013a; Baroni et al., 2014; Levy et al., 2014). The outputs of this model are vector representations that maximize the chance of correctly predicting the context of a word.

#### 3.1.1. Model

We train word2vec on English and Korean Wikipedia dumps (accessed March 2015 and December 2015, respectively) using negative subsampling to generate vector representations of the words in the dataset. We train twelve models with different settings per language; the varied parameters were embedding dimensions (*d* = 300, 600, 1000), window size (*w* = 4, 10) and objective function [continuous bag of words (CBOW) or skip-gram]. Our model setup and measure designs are adapted from the automatic semantic analysis of Wolters et al. (2016). Although Wolters et al. (2016) present both semantic and prosodic levels of analyses, we aim to provide a more focused, expansive discussion on the semantic analysis in this article.

#### 3.1.2. Switch Measures

We design various measures based on the properties of cosine similarity (Equation 3) between two word vectors $\vec{w_{i}}$ and $\vec{w_{j}}$ to identify switches.

(3) $$\begin{array}{l} sim\left(\vec{w_{i}},\vec{w_{j}}\right)=\frac{\vec{w_{i}}\cdot \vec{w_{j}}}{∥\vec{w_{i}}∥\;∥\vec{w_{j}}∥} \end{array}$$

We propose three measures for determining cluster boundaries (i.e., the location of switches).

**Figure 1:**
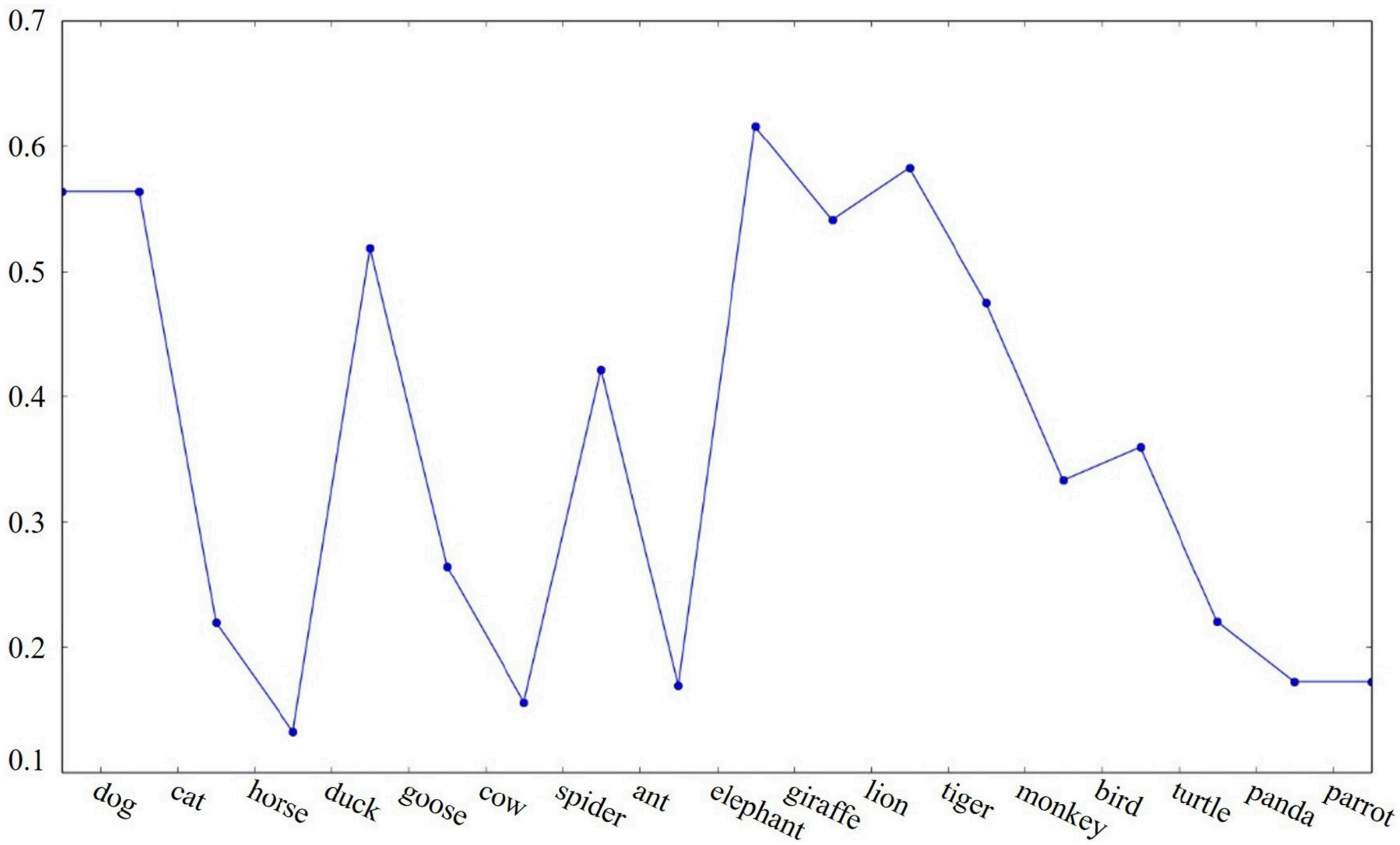
Example of cosine similarity between adjacent words in a semantic fluency sequence.

*Threshold cutoff* : Cluster boundaries are marked where the cosine similarity between two adjacent words falls below a threshold. We test two threshold values that were derived from the dataset, namely the median and the 25th percentile of all cosine similarity values between adjacent pairs of words. As the median (≈ 0.38) is greater than the 25th percentile (≈ 0.25), setting the threshold to the median value marks a cluster boundary between goose and cow in the example sequence shown in Figure 1, whereas the 25th percentile threshold does not.

*Sharp change*: Cluster boundaries are marked where the change in cosine similarity between two adjacent words deviates sharply (twice as large) from the average similarity change between words in the current cluster.

*Inter-group similarity*: Cluster boundaries are marked where the inter-group similarity of the current cluster after the previous switch falls below random inter-group similarity. Inter-group similarity in a cluster *c* of size *n* is defined as the average cosine similarity between all possible pairs of words *w*_*i*_, *w*_*j*_ within *c*.

(4) $$inter(c)=\;\frac{\sum_{i=1}^{n-1}\sum_{j=i+1}^{n}sim(w_{i},w_{j})}{nC_{2}}$$

A meaningful cluster (i.e., a set of semantically related words) would have a higher inter-group similarity compared to a set of randomly selected words. Based on this intuition, we calculated the random inter-group similarity that will serve as the threshold of unrelatedness (thus, a cluster boundary) by applying Equation 4 to a set of *k* words picked at random from the database. To obtain a reliable value, we ran the random selection 1,000 times for 2 ≤ *k* ≤ 5. From these 4,000 random inter-group similarity values, we tested the median and the 75th percentile values to determine cluster boundaries.

The following are the cluster boundaries (marked with “|”) of the example category fluency sequence given in Figure 1 identified by the three different algorithms illustrated above:

*Threshold cutoff* : dog cat | horse | duck goose | cow | spider ant | elephant giraffe lion tiger | monkey | bird turtle | panda | parrot
*Sharp change*: dog cat | horse | duck goose | cow | spider ant | elephant giraffe lion tiger monkey bird turtle | panda | parrot
*Inter-group similarity*: dog cat horse duck goose cow spider | ant | elephant giraffe lion tiger monkey bird turtle panda parrot

### 3.2. Backlink Model

The results of Pakhomov et al. (2012, 2014) show that taxonomic information, such as that which can be derived from WordNet, is a useful analysis tool for semantic fluency data. However, WordNet contains manually curated sets of relations and is not available for many languages. We propose extracting relevant information about relationships between concepts from a resource that is available in over 100 languages, Wikipedia. In addition to having the advantage of larger individual language-level coverage than WordNet, the Backlink method also produces switch counts that correlate better with manual switch counts than a WordNet-based method (see section 4 and Table 5).

#### 3.2.1. Model

The Backlink model is based on a simple intuition that an informative Wikipedia article about a single focused topic represents a potential context for a cluster. To illustrate, an article that explains the “Chinese zodiac”, a frequently occurring theme in Korean folk culture (and several other East Asian cultures), would contain names of the symbolic animals (e.g., *rat, ox, tiger, rabbit*, etc.) that refer to the twelve animal gods. If we examine the linked structures of such articles, we will be able to automatically infer clusters and could even discover clusters not captured by existing manual annotation protocols.

**Figure 2:**
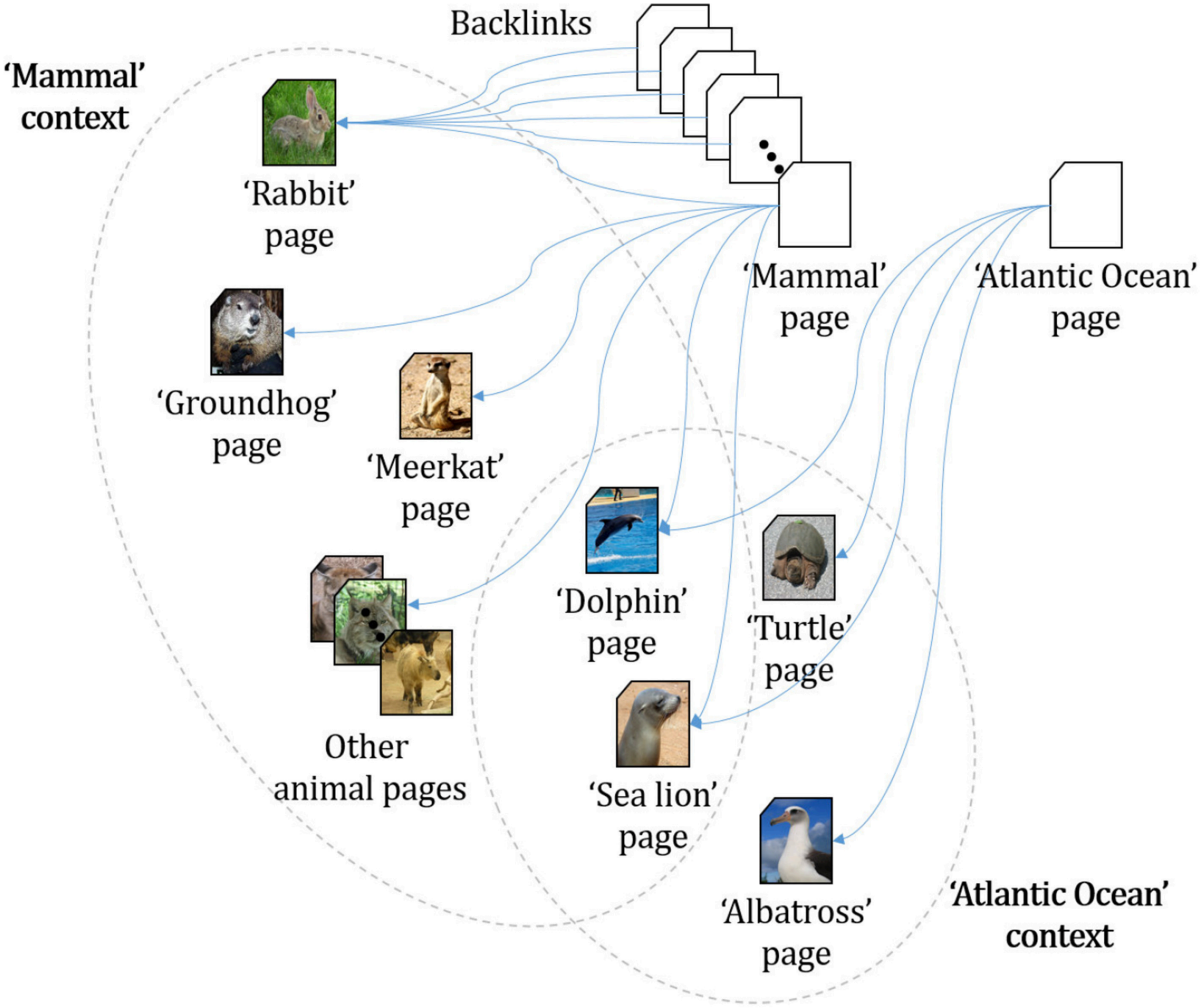
Example of a backlink relation^2^.

To implement this automatic analysis of linked documents, we use the *backlink* information provided by Wikipedia. Every article in Wikipedia includes information about what other pages link back to the current page of interest. For instance, the English Wikipedia page “Rabbit” is backlinked to pages, such as “Mammal”, “Meat”, and “Burrow”. In the “Burrow” article, there are links to other animal articles, such as “Groundhog”, “Mole”, and “Meerkat”. This implies that “Rabbit”, “Groundhog”, “Mole”, and “Meerkat” form a cluster via the context “Burrow” (illustrated in Figure 2). Based on this idea, our automatic analysis algorithm identifies cluster boundaries as follows (details are simplified):

1. Retrieve the list of documents that backlink to a word *w*_*n*_ in a semantic fluency sequence.
2. Retrieve the list of documents that backlink to the subsequent word, *w*_*n*+1_.
3. Find the intersection *I*_*n, n*+1_ of the two lists. The number of documents in the intersection |*I*_*n, n*+1_| represents the shared number of contexts.
4. If |*I*_*n, n*+1_| is below a threshold θ, add a cluster boundary before the subsequent word.

The threshold θ of the shared context size is expected to differ according to the structure of the specific resource that the backlink information is being extracted from. In the case of Wikipedia, languages with more articles would have a higher θ, as they have a larger number of “generic” context articles that do not contribute much information toward detecting significant semantic connections. For instance, pages, such as *List of animal names, List of animals by common name, List of English animal nouns* link to almost all animal documents and therefore are not informative contexts. In our implementation, we heuristically find θ that maximizes the absolute value of the Spearman correlation coefficient between the produced switch counts and age. The values of θ we use are 50 for English, and 1 for Korean. This discrepancy in θ is consistent with the large difference between English and Korean Wikipedia article counts (over 5 million and around 0.35 million, respectively).

#### 3.2.2. Discussion

The Backlink model has a comparatively larger contextual window as it captures inter-document connections between words. The word2vec architecture used to train the VSM uses a limited window of words (4 or 10 in our setting and often fewer than 10 in practical usages) before and after the word of interest (Mikolov et al., 2013a), and thus might not be able to encode document-level information. Like the VSM, the Backlink model can be easily adapted to different languages and cultures by varying the source data (e.g., Wikipedia link structures in different languages).

A major drawback of the Backlink approach is its dependency on the properties of the selected relational knowledge base. This leaves the out-of-vocabulary issue partially unresolved even though the coverage has been substantially expanded, for gaps in the knowledge base are still unable to be processed. For instance, there are no independent articles for common animal names, such as *hen* and *buffalo* in English Wikipedia. Moreover, some minor adjustments were needed due to Wikipedia's specific way of organizing backlink information. For example, backlinks for several words were not retrieved properly due to redirects or disambiguation pages, which needed to be corrected manually. Note that these structural limitations are resource-dependent limitations rather than algorithmic/methodological limitations.

## 4. Evaluation

An acceptable automation of a manual scoring system for semantic fluency should produce output scores that satisfy the following criteria:

1. Correlate well with the manual scores.
2. Reproduce patterns of fluency performance that are known to be reflected in manual scores.

We address Criterion 1 in section 4.4, where we outline how well the VSM and the Backlink model are able to replicate the number of switches and cluster sizes as determined by the traditional manual scoring method.

For Criterion 2, we examine how well the VSM and the Backlink model capture the variance in participant age, since it has been shown in the literature that manual clustering and switching patterns associate with age (section 4.5). In order to evaluate the automatic measures' sensitivity to group differences rather than scalar values of age, we build a logistic regression classifier that determines the age group (younger vs. older) for both English and Korean category fluency test data (section 4.7.2). We furthermore test whether our method can be successfully applied to a different lexical category, fruit, which is, like animals, a well-populated semantic category (section 4.8).

### 4.1. Baseline

We consider a baseline that uses WordNet similarity to determine switch boundaries instead of Backlink or VSM. We use Wu-Palmer similarity (Wu and Palmer, 1994) based on the distance between two words (concepts) that are represented as nodes in WordNet. For the Korean data, we use a Korean-English synset mapping (Choi et al., 2004) in order to alleviate out-of-vocabulary issues with Korean WordNet. Although we described WordNet as a baseline, we expect it to be a strongly competitive model. One of the core motivations in proposing a Wikipedia-based model is its greater coverage of different languages compared to WordNet, and not because the quality of information captured by Wikipedia is considered superior. It could be the case that the manually-curated relational information contained in WordNet is better suited to determining informative switch boundaries. We initially considered a simpler baseline, such as Pointwise Mutual Information (PMI) calculated on Wikipedia text, but it failed to yield an informative measure due to the lack of observed co-occurrences for many adjacent animal pairs in the data. For instance, we only observed six instances of *dog-cat* co-occurrence in the whole corpus, which was one of the most frequent neighboring animal pairs.

### 4.2. Data

We use two sets of semantic fluency test data produced by English and Korean native speakers. The English data were collected from the studies Wolters et al. (2014) and Iveson (2015), whereas the Korean data were collected by members of the NLP*CL lab, KAIST, South Korea for a study of acoustic and linguistic structure of category fluency data including Wolters et al. (2016). We outline the details of each dataset below.

**Figure 3:**
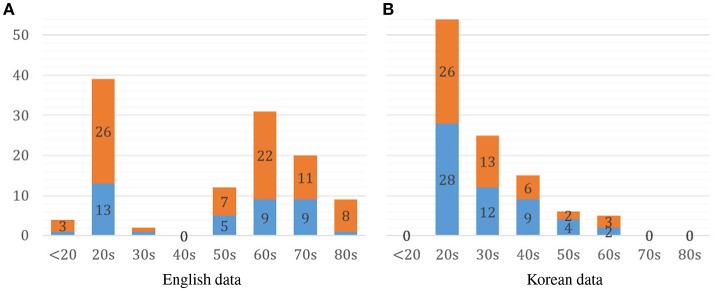
Age and gender distributions of all English and Korean participants (Blue and red bars represent male and female, respectively. The data labels for 1 are omitted). **(A)** Age and gender distribution of English participants. **(B)** Age and gender distribution of Korean participants.

Since the English and Korean data came from two different studies, their age ranges differ. The English data were split into younger and older groups, while the Korean data covered 10-years interval groups of 20s, 30s, 40s, and 50+ (Figure 3). Therefore, when examining the effect of age in the Korean data, we restrict ourselves to two groups: participants aged 20–29 (younger) and participants aged 50+ (older) (Table 1).

**Table 1:** Age and gender distributions of younger and older group English and Korean participants.

	**English**		**Korean**	
	**Male**	**Female**	**Male**	**Female**
Younger	13	26	28	16
(20–29)				
Older	24	48	6	5
(50+)				

#### 4.2.1. English Dataset

The English semantic fluency dataset consists of 117 transcribed semantic fluency sequences produced by English native speakers that were collected as part of the Addenbrooke's Cognitive Examination-Revised (ACE-R) (Mioshi et al., 2006). Participants only performed semantic fluency for the animal category, and responses were recorded by writing them down on the ACE-R form. The mean age was 50.1 years (SD: 23.1, range: 18–84), and 78 (66.7%) were female. The only demographic information collected from the English-speaking participants across all studies was age and gender.

#### 4.2.2. Korean Dataset

Our Korean dataset was collected from 105 Korean native speakers with no self-reported cognitive disorders. The participants were instructed to say out loud as many words as possible that belong to a designated semantic category in 60 seconds, generally following the guidelines of Spreen and Strauss (1998). Participants were asked to perform this task twice using two different semantic categories: animals and fruits (the order between the two categories was randomized). An experimenter used a timer to notify the participants when each task started and ended. These spoken sequences were recorded digitally, and afterwards two Korean native speakers (authors NK and JK; non-participants) transcribed each audio file.

The mean age of the participants was 32.6 years (SD: 11.5, range: 20–64), and 50 (47.6%) were female. Participants' full-time years of school education was 16.0 years (SD: 2.8, range: 9–25).

#### 4.2.3. Manual Clustering and Switching Analysis

We followed the established method of scoring semantic fluency as described in Troyer et al. (1997). Two of the authors (NK and JK) manually annotated each semantic fluency sequence for both the English and Korean datasets. As no detailed protocol exists for switching and clustering annotation exclusively for scoring Korean data, we used an adapted version of the English annotation protocol, following Sa et al. (2011).

We note that importing the English scoring method directly into Korean without any consideration of linguistic and cultural discrepancies might affect our manual scores. Since our adaptable model addresses cultural differences, it is possible that the automatic analysis may outperform the manual one.

Table 2 lists Krippendorff's α for the switch counts and cluster sizes reported by the two annotators. All α values except for the α for median cluster size are above the reliable level (α = 0.8) suggested by Krippendorff (2004). The α for median cluster size in English (α = 0.736) is above the reliability level that can be used to draw tentative conclusions (α = 0.667), but α for Korean median cluster size (α = 0.638) is below this level. As previously noted, this could reflect the disagreement due to the annotators having to apply the English protocol to Korean data. That is, the annotators had to make subjective decisions to find English words in the guidelines that correspond to the Korean words in the data. Since all other values of α are sufficiently large, we accept one annotator (JK)'s results as the gold standard and use these values consistently throughout our discussion (referred to as manual scores henceforth). We report correlations with model scores with all measures in **Table 5** for completeness, but one should consider the low α for Korean median cluster size. Our choice of the gold standard annotator is consistent with Wolters et al. (2016).

**Table 2:** Inter-annotator agreements for manual analysis.

**Language**	**Switches**	**Cluster size**		
		**Mean**	**Median**	**Max**
English	0.962	0.895	0.736	0.888
Korean	0.972	0.866	0.638	0.937

### 4.3. VSM Model Selection

We trained twelve English and twelve Korean VSMs with different hyperparameter combinations as discussed in section 3.1.1. For each language, we chose the model that had the highest correlation with manual switch counts. Spearman's rank order correlation was used to compute correlation coefficients. We selected the *Threshold cutoff* method with the median threshold as our criterion for determining automatic switch counts, as it performed best in preliminary analyses, and the absolute values produced are close to the manually established values.

All English models (*p* < 0.00001 or better) and all Korean models (*p* < 0.001 or better) yielded significant correlations with manual switch counts. For English, the best model was the skip-gram model with *d* = 300, *w* = 4 (ρ = 0.657), and for Korean, the best model was the CBOW model with *d* = 1000, *w* = 4 (ρ = 0.572). All reported results henceforth are from these settings, unless stated otherwise. For Korean, we found that a second model, KDiff (CBOW, *d* = 600, *w* = 10), yielded worse correlation with manual switches (ρ = 0.525, *p* < 0.001), but performed better in the final evaluation (**Tables 9, 12**). This might be due to the transferability issue noted in section 2, which we discuss further in section 5. Results from the KDiff model are additionally reported along with the results from the two main models, and discussed where relevant.

### 4.4. Correlation Between Manual and Automatic Analysis

Among the three VSM switch identification strategies proposed in section 3.1.2, the median *Threshold cutoff* correlated best with the results of the manual analysis both for English (Spearman's ρ = 0.657, *p* < 0.001) and for Korean (ρ = 0.572, *p* < 0.001) data. Thus, we use the median *Threshold cutoff* consistently to mark switch boundaries when reporting VSM results. Full results for all three proposed measures are given in Table 3. Furthermore, we note that the tendency of older participants to switch less often than younger participants (Troyer, 2000) is generally well-captured by the median *Threshold cutoff* (Table 4).

**Table 3:** Correlation (Spearman's ρ) between switch counts determined by VSM and manual scoring.

**Language**	**Age groups**	***Threshold cutoff***	***Sharp changes***	***Inter-group similarity***
English	all	0.657^***^ (median)	0.342^***^	−0.054 (median)
		0.390^***^ (25th)		−0.010 (75th)
	younger	0.750^***^ (median)	0.285	−0.005 (median)
		0.333^*^ (25th)		−0.046 (75th)
	older	0.562^***^ (median)	0.305^**^	−0.236^*^ (median)
		0.434^***^ (25th)		−0.275^*^ (75th)
Korean	all	0.572^***^ (median)	0.565^***^	0.173 (median)
		0.373^***^ (25th)		0.499^***^ (75th)
	younger	0.598^***^ (median)	0.604^***^	0.263 (median)
		0.482^***^ (25th)		0.587^***^ (75th)
	older	−0.012 (median)	0.535	−0.163 (median)
		0.021 (25th)		0.074 (75th)
Korean (KDiff)	all	0.525^***^ (median)	0.518^***^	0.144 (median)
		0.327^***^ (25th)		0.429^***^ (75th)
	younger	0.560^***^ (median)	0.544^***^	0.224 (median)
		0.417^**^ (25th)		0.499^***^ (75th)
	older	−0.026 (median)	0.664^*^	−0.141 (median)
		0.129 (25th)		−0.069 (75th)

* *(p < 0.05*,

** *p < 0.01*,

*** *p < 0.001)*.

**Table 4:** Average switch counts for each scoring metric according to age groups.

**Language**	**Age groups**	**Manual**	**VSM**					**Backlink**
			***Threshold cutoff***		***Sharp***	***Inter-group similarity***		
			**25th**	**median**	***change***	**75th**	**median**	
English	all	9.786	4.974	9.949	5.658	0.983	0.308	12.427
	younger	10.949	5.256	10.513	6.051	0.846	0.385	13.564
	older	9.097	4.861	9.639	5.431	1.111	0.292	11.732
Korean	all	10.657	5.152	10.381	10.657	10.552	4.219	7.543
	younger	10.944	5.074	10.519	10.889	10.667	4.185	8.056
	older	9.927	6.091	10.000	9.636	10.000	5.273	5.000
Korean (KDiff)	all	10.657	5.181	10.381	10.438	9.533	3.695	7.543
	younger	10.944	5.204	10.278	11.093	9.519	3.741	8.056
	older	9.927	6.091	9.909	9.273	9.455	4.818	5.000

*Korean and Korean (KDiff) only differ by their VSM switch counts*.

The Backlink method also produced switch counts that correlated well with manual switches, in both English (ρ = 0.618, *p* < 0.001) and Korean (ρ = 0.554, *p* < 0.001). As can be seen from Table 5, both models yield switch counts that show higher correlations with the manual counts, compared to the WordNet-based models.

**Table 5:** Correlation between switch counts and cluster sizes determined by manual scoring and by the selected best settings for automatic scoring algorithms.

**Language**	**Model**	**Switch count**	**Mean cluster size**	**Median cluster size**	**Max cluster size**
English	WordNet	0.561^***^(9.846)	0.402^***^(2.064)	0.454^***^(1.581)	0.113 (4.871)
	VSM	0.657^***^(9.949)	0.568^***^(1.998)	0.355^***^(1.500)	0.221^*^(4.641)
	Backlink	0.618^***^(12.427)	0.242^**^(2.068)	0.149 (1.949)	0.261^**^(3.641)
Korean	WordNet	0.535^***^(10.343)	0.425^***^(2.075)	0.323^***^(1.452)	0.196^*^(5.257)
	VSM	0.572^***^(10.381)	0.141 (2.000)	0.140 (1.205)	0.258^**^(6.057)
	VSM (KDiff)	0.525^***^(10.381)	0.105 (2.026)	0.027 (1.262)	0.219^*^(5.943)
	Backlink	0.554^***^(7.543)	−0.0153 (3.488)	0.072 (2.871)	0.031 (6.990)

Values inside parentheses denote the average of the actual values

* *(p < 0.05*,

** p < 0.01, and

*** *p < 0.001)*.

### 4.5. Age

#### 4.5.1. Manual Switch Counts

The normative dataset reported in Troyer (2000) suggests that the switch count calculated by the manual scoring procedure discussed in section 4.2.3 is reversely associated with age (i.e., the older the individual, the fewer the number of switches). Although the English manual switch counts reflect this finding (ρ = −0.318, *p* < 0.001), we found no significant reverse correlation in Korean (ρ = −0.119, *p* = 0.228). This shows the limited transferability of the manual protocol across languages, as discussed in sections 2 and 4.2.3.

#### 4.5.2. Automatic Switch Counts

The correlation between age and VSM switch counts for Korean were not significant (ρ = −0.104, *p* > 0.05) whereas English was (ρ = −0.275, *p* < 0.01). This aligns with the pattern we observed in manual scores. The Backlink model showed a comparatively more stable prediction across the two languages. The switch counts correlated negatively with age in both English (ρ = −0.354, *p* < 0.001) and Korean (ρ = −0.186, *p* = 0.057), although only the English data reached statistical significance. Switch counts from the WordNet model showed similar results to VSM, with English switches correlating negatively with age (ρ = −0.353, *p* < 0.001) but no significant association between Korean switches and age (ρ = 0.063, *p* > 0.05). These results are summarized in Table 6.

**Table 6:** Correlation between age and switch counts produced by different models.

**Language**	**Model**	**Age-switch count correlation **(ρ)****
English	Manual	−0.318^***^
	WordNet	−0.353^***^
	VSM	−0.275^**^
	Backlink	−0.354^***^
Korean	Manual	−0.119
	WordNet	0.063
	VSM	−0.104
	VSM (KDiff)	−0.023
	Backlink	−0.186 (*p* = 0.057)

* *p < 0.05*,

** p < 0.01, and

*** *p < 0.001*.

### 4.6. Gender and Education

Although demographic information other than age, namely gender and also years of education for Korean participants, was collected, these factors did not have significant effect on any of the discussed measures in either English or Korean (Table 7), with the exception of Backlink max cluster size. Gender differences were tested using the Mann-Whitney-Wilcoxon test and correlations with years of education were tested using Spearman's rank-order correlation.

**Table 7:** Influence of gender and education on manual semantic fluency scores.

**Language**	**Factors**	**Test**
English	Gender-Manual	*U* = 1380.5
	Gender-Backlink switch count	*U* = 1370.5
	Gender-Backlink mean cluster size	*U* = 1631
	Gender-Backlink median cluster size	*U* = 1536
	Gender-Backlink max cluster size	*U* = 1918.5^*^
	Gender-VSM switch count	*U* = 1490
	Gender-VSM mean cluster size	*U* = 1528
	Gender-VSM median cluster size	*U* = 1487
	Gender-VSM max cluster size	*U* = 1549.5
Korean	Gender-Manual	*U* = 1496
	Gender-Backlink switch count	*U* = 1568
	Gender-Backlink mean cluster size	*U* = 1163
	Gender-Backlink median cluster size	*U* = 1347
	Gender-Backlink max cluster size	*U* = 878.5^**^
	Gender-VSM switch count	*U* = 1271.5
	Gender-VSM mean cluster size	*U* = 1574.5
	Gender-VSM median cluster size	*U* = 1576.5
	Gender-VSM max cluster size	*U* = 1295
	Gender-VSM (KDiff) switch count	*U* = 1248
	Gender-VSM (KDiff) mean cluster size	*U* = 1603.5
	Gender-VSM (KDiff) median cluster size	*U* = 1571.5
	Gender-VSM (KDiff) max cluster size	*U* = 1339
	Education-Manual	ρ = −0.041
	Education-Backlink switch count	ρ = 0.079
	Education-Backlink mean cluster size	ρ = −0.103
	Education-Backlink median cluster size	ρ = −0.121
	Education-Backlink max cluster size	ρ = 0.056
	Education-VSM switch count	ρ = 0.107
	Education-VSM mean cluster size	ρ = −0.069
	Education-VSM median cluster size	ρ = −0.118
	Education-VSM max cluster size	ρ = 0.034
	Education-VSM (KDiff) switch count	ρ = 0.147
	Education-VSM (KDiff) mean cluster size	ρ = −0.105
	Education-VSM (KDiff) median cluster size	ρ = −0.010
	Education-VSM (KDiff) max cluster size	ρ = 0.002

* *p < 0.05*,

** p < 0.01, and

*** *p < 0.001*.

### 4.7. Backlink-VSM

We jointly consider the outputs of Backlink and VSM models for an integrated analysis. We first conduct linear regression analyses that use measurements from both the VSM and the Backlink model as predictors of age, and then use logistic regression analyses to examine the sensitivity of the models to different age groups (younger vs. older). As well as the switch counts, we also include cluster sizes as predictors following the literature (Troyer et al., 1997; Methqal et al., 2019) (see Table 8 for the full list).

**Table 8:** List of proposed predictors from the VSM and the Backlink model.

**Model**	**Variable**
Backlink	Switch count
	Mean cluster size
	Median cluster size
	Max cluster size
Vector Space	Switch count
	Mean cluster size
	Median cluster size
	Max cluster size

#### 4.7.1. Predicting Age With an Integrated Model

Since many of the proposed predictors are expected to be collinear, we conducted a Variance Inflation Factor (VIF) analysis to exclude potentially multicollinear predictors. Table 9 shows the regression models fit with predictors with VIF >10 removed from the full model. We find that the Backlink + VSM models are statistically significant for English, but not for Korean. However, Backlink + VSM using KDiff VSM is significant. This suggests that a better-performing model is not necessarily equivalent to the best-correlating model with manual scores if the manual scores are calculated using a ported protocol. Further discussion is made in section 5.

**Table 9:** Linear regression using features from the VSM and the Backlink model as predictors of age.

**Model**	**Predictors**	**B**
English (Backlink + VSM only) (*R*^2^ = 0.191, *F*(6, 110) = 4.314, *p* < 0.001)	Backlink switch count	−2.684^***^
	Backlink mean cluster size	−21.508
	Backlink median cluster size	−10.051
	Backlink max cluster size	2.289
	VSM median cluster size	8.357^*^
	VSM max cluster size	1.706
English (Backlink + VSM + WC & Rep) (*R*^2^ = 0.240, *F*(8, 108) = 4.271, *p* < 0.001)	Backlink switch count	−7.287^***^
	Backlink mean cluster size	−35.885^*^
	Backlink median cluster size	−5.935
	Backlink max cluster size	−0.908
	VSM median cluster size	8.038^*^
	VSM max cluster size	1.205
	Unique word count	3.179^*^
	Repetition	10.058^*^
Korean (Backlink + VSM only) (*R*^2^ = 0.087, *F*(6, 98) = 1.559, *p* = 0.167)	Backlink switch count	−0.471
	Backlink max cluster size	0.640
	VSM switch count	−0.241
	VSM mean cluster size	3.306
	VSM median cluster size	−5.159
	VSM max cluster size	−0.545
Korean (Backlink + VSM + WC & Rep) (*R*^2^ = 0.278, *F*(8, 96) = 4.621, *p* < 0.001)	Backlink switch count	−0.431
	Backlink max cluster size	0.279
	VSM switch count	−0.714
	VSM mean cluster size	−2.176
	VSM median cluster size	−2.201
	VSM max cluster size	−0.284
	Unique word count	0.118
	Repetition	5.224^***^
Korean (KDiff) (Backlink + VSM only) (*R*^2^ = 0.123, *F*(6, 98) = 2.286, *p* < 0.05)	Backlink switch count	−0.768
	Backlink max cluster size	0.348
	VSM switch count	0.032
	VSM mean cluster size	5.648
	VSM median cluster size	−7.451^*^
	VSM max cluster size	−0.642
Korean (KDiff) (Backlink + VSM + WC & Rep) (*R*^2^ = 0.294, *F*(8, 96) = 4.993, *p* < 0.001)	Backlink switch count	−0.491
	Backlink max cluster size	0.207
	VSM switch count	0.028
	VSM mean cluster size	2.546
	VSM median cluster size	−4.466
	VSM max cluster size	−0.241
	Unique word count	−0.278
	Repetition	4.449^***^

* *p < 0.05*,

** p < 0.01, and

*** *p < 0.001*.

Table 9 also reports contributions of two predictive factors of age in addition to the measures obtained from the VSM and the Backlink model: unique word counts and perseveration errors (i.e., repetition). Unique word counts, which are the standard analysis metric for verbal fluency tests, have been found to decline with age (Lezak, 2004). Our English and Korean datasets both demonstrated this tendency as displayed in the word count statistics given in Table 10.

**Table 10:** Unique word counts for English and Korean data grouped by age.

**Language**	**Age groups**	**Unique word count**	
		**Mean (SD)**	**Range**
English	all	20.69 (4.69)	[11, 35]
	younger	21.71 (4.87)	[12, 30]
	older	20.08 (4.56)	[11, 35]
Korean	all	21.03 (5.91)	[8, 42]
	younger	21.69 (6.05)	[8, 42]
	older	16.55 (4.01)	[11, 24]

The significance of perseveration errors, which was also acknowledged by March and Pattison (2006), only emerges for the Korean data. Looking at both datasets, we see that only 19% of the English data had at least 1 instance of a perseveration error, whereas 39% of the Korean data contained such errors. This may be due to administration and transcription practices. The Korean data were transcribed from audio recordings after data collection, whereas the English data were transcribed as participants spoke, and there are no audio recordings. There is no requirement to record perseveration errors when administering semantic fluency as part of the ACE-R.

#### 4.7.2. Predicting Age Groups With an Integrated Model

We further evaluate our integrated model by its ability to distinguish between the younger (20–29) and older (50 and over) age groups using logistic regression. We use all of the four proposed predictors (switch count, mean/median/max cluster sizes) for English models. We removed median cluster size from Korean models and used only three (switch count, mean/max cluster sizes) because the particular predictor prevented the model from fitting correctly^3^. In building the composite Backlink + VSM models, we always use the same number of predictors as the singleton models (either Backlink or VSM) for fair comparison. The results are reported in Table 11.

**Table 11:** Performance of the integrated model for the younger-older group distinction using animal fluency.

**Language**	**Model**	**χ^2^ (df)**	**Accuracy (%)**	**P (%)**	**R (%)**
English	Manual	15.068^**^(4)	71.1	71.7	91.7
	Majority class	-	64.9 (39:72)	-	-
	WordNet	7.977^**^(4)	68.5	71.3	86.1
	VSM	4.120 (4)	65.8	65.5	100
	Backlink	9.769^*^(4)	70.3	72.4	87.5
	Backlink + VSM	12.413^*^(4)	72.1	74.7	86.1
	Backlink + VSM + WC + Rep.	25.097^***^(6)	73.9	72.6	95.8
Korean	Manual	5.085 (4)	84.6	100	9.1
	Majority class	-	83.1 (54:11)	-	-
	WordNet	13.078^**^(3)	86.2	62.5	45.5
	VSM	4.441 (3)	86.2	100	18.2
	VSM (KDiff)	5.071 (3)	86.2	100	18.2
	Backlink	9.583^**^(3)	84.6	66.7	18.2
	Backlink + VSM	9.784^**^(3)	87.7	100	27.3
	Backlink + VSM (KDiff)	10.560^**^(3)	87.7	100	27.3
	Backlink + VSM + WC + Rep.	26.796^***^(5)	90.8	85.7	54.5
	Backlink + VSM (KDiff) + WC + Rep.	27.102^***^(5)	90.8	85.7	54.5

* *(p < 0.05*,

** p < 0.01, and

*** *p < 0.001)*.

Our most trivial baseline is the accuracy when all datapoints are classified into the majority group (“majority class”). For example, for English majority class accuracy is when all 111 samples are predicted to be over 50; in this case, 72 instances will be marked as correct, and thus the accuracy is 72/111 ≈ 64.9%. We also provide comparisons with models using predictors derived from manual and WordNet switches.

All tested models outperform majority class. However, for Korean, the improvement over the majority class baseline using manual scoring is more modest than for English, where manual scoring (unsurprisingly) shows strong performance. The integrated model performs better than the manual scoring model in both languages. Especially in English, only the composite Backlink + VSM model yields accuracy above manual scoring even with the same number of predictors. No singleton model, including WordNet, outperformed the manual scoring model in English. In Korean, for which manual scoring is a comparatively weaker model, some singleton models do outperform this. However, the best performance is still achieved by an integrated model. These results align with our original design goal that the VSM and the Backlink model would complement each other to make better predictions.

### 4.8. Generalization to Different Category

We conducted an additional analysis to test the adaptability of our integrated model across semantic categories. Prior works, such as March and Pattison (2006) have highlighted the need for conducting semantic fluency tests with multiple categories for a more complete picture of cognitive processes, as numerous category-specific effects have been reported. The most commonly used semantic categories in clinical assessment are animals and supermarket items, and these two domains have relatively well-established scoring protocols. However, for other popular domains, such as fruit, standardized scoring protocols are not available, making the scoring process reliant on the arbitrary decisions of individual experimenters. There is also likely to be considerable cultural variation in the fruits mentioned.

A robust automation should be able to deal with this issue, being able to draw consistent distinctions between age groups across category. We test whether our integrated model can achieve this robustness when applied to data from a different semantic domain. The model used in section 4.7.2 was directly applied to the Korean fruit category fluency sequences produced by the same 105 Korean subjects (with only minor manual adjustments mentioned in section 3.2.2). Table 12 shows that the patterns we saw in the animal category do generalize. We could achieve accuracy above the majority class baseline with all tested models except singleton VSM, with the Backlink + VSM model leading to better performance than singleton Backlink or VSM models with the same number of predictors. We also note that the WordNet model failed to mark informative switch boundaries (e.g., no switch boundaries were found in a sequence) due to out-of-vocabulary issues. This adds further support for the cross-domain generalizability of our model.

**Table 12:** Performance of the integrated model for the younger-older group distinction using fruit fluency.

**Language**	**Model**	**χ^2^ (df)**	**Accuracy (%)**	**P (%)**	**R (%)**
Korean	Majority class	-	83.1 (54:11)	-	-
	VSM	7.121 (4)	81.5	40.0	18.2
	VSM (KDiff)	5.832 (4)	84.6	66.7	18.2
	Backlink	22.291^***^(4)	87.7	100	27.3
	VSM + Backlink	22.783^***^(4)	89.2	83.3	45.5
	VSM (KDiff) + Backlink	20.477^***^(4)	89.2	100	36.4
	VSM + Backlink + WC + Rep.	26.894^***^(6)	90.8	85.7	54.5
	VSM (KDiff) + Backlink + WC + Rep.	32.215^***^(6)	93.9	88.9	72.7

* *(p < 0.05*,

** p < 0.01, and

*** *p < 0.001)*.

## 5. Discussion

Our integrated model for scoring semantic fluency is capable of distinguishing younger and older age groups by measures obtained from the VSM and the Backlink model. From analyzing the results of the younger/older classification, we can conclude that the two different models complementarily contribute to accurate predictions of the participants' age groups. The predictive power of the integrated model is even stronger than a model based on the traditional scoring method. Positive results as such were observed across linguistic/cultural domains (English-Korean) and across semantic domains (animal-fruit), which gives us promising prospects for an automatic model with high adaptability. We also highlight the fact that both Backlink and VSM were built from data extracted from the same source: Wikipedia.

We also note that for Korean, the VSM model that produces switch counts that best correlate with the manual scores was sometimes less effective in predicting participant age compared to an alternative model (KDiff). The main Korean model replicates the patterns shown by manual scoring, and therefore satisfies Criterion 1 in section 4. However, the transferability issue in manual annotation protocols across languages results in a comparatively less effective model in predicting participant age (Criterion 2). Results using KDiff VSM demonstrates that our proposed method has the capacity to yield a more generalizable model that satisfies Criterion 2, although it correlates to a lesser degree with the manual scoring model. This potentially calls for a different model optimization strategy for languages that do not have an established manual scoring protocol.

The presented results demonstrate that our Backlink-VSM outperforms the standalone VSM proposed for the semantic analysis of category fluency data in Wolters et al. (2016). This improvement was achieved by introducing the Backlink model that captures relational information potentially overlooked by the VSM, and also by experimenting with additional metrics other than the number of switches (e.g., average cluster size, unique cluster counts, cluster overlap counts, etc.) noted by prior research (Abwender et al., 2001; Lanting et al., 2009). The addition of Backlink was especially crucial for adaptability to Korean (see Tables 11, 12), the comparatively lower-resource language in our experiments. Moreover, we have demonstrated that the automatic analysis that found significant results in Wolters et al. (2016), which used a smaller set of data collected from 20 Korean speakers with an age range between 18 and 27 years, continues to produce significant results when extensively applied to larger (dataset size) and broader (language, domain, age range variations) groups of participants.

While Wikipedia is not as well-curated as a psycholinguistic database, it is substantially larger, and more likely to contain the words produced by participants. This is particularly relevant in cases where a person has a particularly deep knowledge of the semantic field. For example, in the English dataset, a person well-versed in ornithnology produced over twenty species of birds in answer to the original stimulus.

### 5.1. Limitations and Future Work

Even though our preliminary results are satisfactory, we note some experimental limitations that need to be considered in future work. Most important, the sample size of the Korean older group was small (11 participants), which caused an imbalance in the ratio between the younger and older groups. This resulted in a generally higher accuracy of binary classification for the Korean integrated model. Thus, the accuracy of the evaluation results should be understood in comparison to the performance of the manual scoring model rather than be taken at face value. In future studies, our priority is to recruit more Korean participants from the older age group. Additionally, we will reinforce the validation of our integrated model by conducting cross-validation with the added datapoints.

The Backlink-VSM model itself bears several limitations. As discussed in section 3.2.2, the performance of the Backlink model is expected to be dependent on the structure and richness of the selected knowledge base. We have shown that our model outperforms a WordNet similarity-based model, but this potential effect of varying the data source should be tested further. We could also reinforce the Backlink extraction algorithm itself, using recent developments, such as RelFinder (Heim et al., 2009). Adding a function that systematically resolves ambiguity could also be useful in reducing manual adaptations in the application of our algorithm. Furthermore, there exist strong predictors of age that we have identified during our experiments but did not include for the sake of consistency, such as Backlink overlap counts. If we were to build a more applied system with focus on optimizing for accuracy, these predictors should be considered.

In VSM, the three cosine similarity-based algorithms that determine clustering and switching may not necessarily be the optimal solutions. It might be possible to develop measures that yield even better results, that need not rely on cosine similarity. Our cosine similarity measures are also open to improvements–for example, it would be interesting to let the threshold in the *Threshold cutoff* measure be set according to the average cosine similarity of each participant, rather than the whole dataset. Another point to note is that the current VSM measures are not sensitive to cluster overlaps unlike the manual scoring method; adding an algorithm that detects overlaps could further improve the agreement between the automatic and gold standard scoring methods. We should also review the implications of category fluency conducted in different domains more carefully and reflect this in future work, as it has been suggested that different semantic domains could tap into different cognitive functions (March and Pattison, 2006). Furthermore, VSM performance is dependent on the size of the training data; the Korean vector space model not being able to make significant contributions to the integrated model in comparison to its English counterpart may be attributed to this factor.

We also acknowledge the inherent limitations of using a model derived from a database that is not specifically constructed for representing semantic relations. The semantics relations captured by our model could potentially confound finer-grained types of relations that hold between lexical items; for instance the Backlink model would not necessarily tease apart co-hyponymy and functional semantic relations.

As noted in section 2.2, most prior attempts at automating semantic fluency scoring have devised novel sets of metrics, rather than designing metrics that closely follow the established switching and clustering-based manual scoring (Prescott et al., 2006; Pakhomov et al., 2012; Sung et al., 2012; Nicodemus et al., 2014). Each of these works claims that their new metrics improve the drawbacks of traditional assessment methodology, which we should take into account and possibly incorporate in design improvements.

Moving beyond the scope of this paper, a more fundamental question regarding the original aims of verbal fluency tests remains to be answered; can our automated model be used to compute scores that reliably draw the distinction between healthy aging and cognitive impairment?

## 6. Conclusion

We designed and tested a scoring model of semantic fluency. Based on prior work reporting the effect of aging on clustering and switching in neurotypical participants, we developed an automated version of the established scoring protocol that successfully distinguishes between younger and older age groups. Our automation outperforms the manual scoring model and a WordNet-based model in all experiments for both English and Korean, and furthermore achieves generalizability across semantic domains. At the same time, our method eliminates the need for a hand-coded fixed taxonomy traditionally used for determining semantic clusters. Instead, our proposed method exploits information extracted from accessible public resources to train a more adaptable but inexpensive scoring model. The evaluation results of the English model presented in this study demonstrate that our idea of combining the vector space model and the Backlink model that theoretically complement each other does indeed yield improved performance. Although the Korean model did not perform as well as the English model, its comparison with the manual model's accuracy looks promising. We believe future research with a larger and more demographically refined dataset would enable us to further improve our method's adaptability across languages and domains. Extending the application of the system to actual patient data is also within the scope of our future work.

## Ethics Statement

This study was carried out in accordance with the recommendations of South East Scotland NHS Ethics Board, Philosophy, Psychology, and Language Sciences Ethics Committee of the University of Edinburgh, and Institutional Review Board of KAIST with written informed consent from all participants. All participants gave written informed consent in accordance with the Declaration of Helsinki. The protocol was approved by South East Scotland NHS Ethics Board, Philosophy, Psychology, and Language Sciences Ethics Committee of the University of Edinburgh, and Institutional Review Board of KAIST.

## Author Contributions

All authors conceived the proposed approach. NK and J-HK designed and performed the evaluations, implemented the proposed models, and analyzed the experimental data. NK and J-HK wrote the first draft of the manuscript with support from JP. MW and SM wrote sections of the manuscript. All authors discussed the results, contributed to the final manuscript and approved the submitted version.

### Conflict of Interest Statement

The authors declare that the research was conducted in the absence of any commercial or financial relationships that could be construed as a potential conflict of interest.

## Footnotes

^1^This is in line with the failure of Pointwise Mutual Information to serve as a reasonable baseline; see section 4.1.

^2^LeBlanc, C. (2007). groundhog. April 8, 2007 via Flickr, used under CC BY, Creative Commons Attributions. https://www.flickr.com/photos/99345230@N00/450155256 Murphy, K. (2011). Meerkat. August 10, 2011 via Flickr, used under CC BY, Creative Commons Attributions. https://www.flickr.com/photos/33957625@N06/6645376763 Goebel, G. (2008). Ybkug 2b. April 9, 2008 via Flickr, used under CC BY, Creative Commons Attributions. https://www.flickr.com/photos/37467370@N08/7596610600 Goebel, G. (2012). Ybtak 2b. July 20, 2012 via Flickr, used under CC BY, Creative Commons Attributions. https://www.flickr.com/photos/37467370@N08/7612070136 Goebel, G. (2013). Yblyx 1b. August 27, 2013 via Flickr, used under CC BY, Creative Commons Attributions. https://www.flickr.com/photos/37467370@N08/16010887138 Goebel, G. (2014). Ybbun 4b. June 15, 2014 via Flickr, used under CC BY, Creative Commons Attributions. https://www.flickr.com/photos/37467370@N08/16239252022 Caranta, A. (2009). Dolphin jump. October 3, 2009 via Flickr, used under CC BY, Creative Commons Attributions. https://www.flickr.com/photos/87204754@N00/3986742924 Orleans, D. (2008). Turtle. June 7, 2008 via Flickr, used under CC BY, Creative Commons Attributions. https://www.flickr.com/photos/9788232@N03/2644894340 Yeomans, S. (2006). Sea lion. January 2, 2006 via Flickr, used under CC BY, Creative Commons Attributions. https://www.flickr.com/photos/36302954@N00/81133167Region, U. P. (2006). Laysan albatross. December 19, 2006 via Flickr, used under CC BY, Creative Commons Attributions. https://www.flickr.com/photos/52133016@N08/5562256437

^3^Also, Korean median cluster size happens to be the only predictor that did not have a reliable inter-annotator agreement (section 4.2.3).

## References

[B1] Abwender D. A. Swan J. G. Bowerman J. T. Connolly S. W. (2001). Qualitative analysis of verbal fluency output: review and comparison of several scoring methods. Assessment. 8, 323–338. 10.1177/107319110100800308 11575625

[B2] Baroni M. Bernardini S. Ferraresi A. Zanchetta E. (2009). The WaCky wide web: a collection of very large linguistically processed web-crawled corpora. Lang. Resour. Eval. 43, 209–226. 10.1007/s10579-009-9081-4

[B3] Baroni M. Dinu G. Kruszewski G. (2014). Don't count, predict! a systematic comparison of context-counting vs. context-predicting semantic vectors, in Proceedings of the 52nd Annual Meeting of the Association for Computational Linguistics (Volume 1: Long Papers), (Baltimore, MD) 238–247.

[B4] Chan A. S. Butters N. Salmon D. P. McGuire K. A. (1993). Dimensionality and clustering in the semantic network of patients with Alzheimer's disease. Psychol. Aging. 8, 411–419. 8216961 10.1037//0882-7974.8.3.411

[B5] Choi K.-S. Bae H.-S. Kang W. Lee J. Kim E. Kim H. (2004). Korean-chinese-japanese multilingual wordnet with shared semantic hierarchy, in Proceedings of the Fourth International Conference on Language Resources and Evaluation (Lisbon), 1131–1134.

[B6] Clark D. G. Kapur P. Geldmacher D. S. Brockington J. C. Harrell L. DeRamus T. P.. (2014). Latent information in fluency lists predicts functional decline in persons at risk for Alzheimer disease. Cortex. 55, 202–218. 10.1016/j.cortex.2013.12.013 24556551 PMC4039569

[B7] Clark D. G. McLaughlin P. M. Woo E. Hwang K. Hurtz S. Ramirez L.. (2016). Novel verbal fluency scores and structural brain imaging for prediction of cognitive outcome in mild cognitive impairment. Alzheimer's Dement. 2, 113–122. 10.1016/j.dadm.2016.02.001 27239542 PMC4879664

[B8] Dubois B. Slachevsky A. Litvan I. Pillon B. (2000). The FAB: a frontal assessment battery at bedside. Neurology. 55, 1621–1626. 10.1212/wnl.55.11.1621 11113214

[B9] Haugrud N. Crossley M. Vrbancic M. (2011). Clustering and switching strategies during verbal fluency performance differentiate alzheimer's disease and healthy aging. J. Int. Neuropsychol. Soc. 17, 1153–1157. 10.1017/S1355617711001196 22014065

[B10] Haugrud N. Lanting S. Crossley M. (2010). The effects of age, sex and alzheimer's disease on strategy use during verbal fluency tasks. Aging Neuropsychol. Cogn. 17, 220–239. 10.1080/13825580903042700 19642046

[B11] Hazin I. Leite G. Oliveira R. M. Alencar J. C. Fichman H. C. Marques P. D. N.. (2016). Brazilian normative data on letter and category fluency tasks: effects of gender, age, and geopolitical region. Front. Psychol. 7:684. 10.3389/fpsyg.2016.00684 27242598 PMC4861882

[B12] Heim P. Hellmann S. Lehmann J. Lohmann S. Stegemann T. (2009). RelFinder: revealing relationships in RDF knowledge bases, in International Conference on Semantic and Digital Media Technologies (Graz: Springer), 182–187.

[B13] Henry J. D. Crawford J. R. Phillips L. H. (2004). Verbal fluency performance in dementia of the Alzheimer's type: a meta-analysis. Neuropsychologia. 42, 1212–1222. 10.1016/j.neuropsychologia.2004.02.001 15178173

[B14] Hills T. T. Jones M. N. Todd P. M. (2012). Optimal foraging in semantic memory. Psychol. Rev. 119, 431–440. 10.1037/a0027373 22329683

[B15] Iveson M. (2015). Goal Maintenance: Examining Capacity, Competition, and Duration, and Their Relation to Intelligence and Processing Speed. PhD thesis, The University of Edinburgh.

[B16] König A. Linz N. Tröger J. Wolters M. Alexandersson J. Robert P. (2018). Fully automatic speech-based analysis of the semantic verbal fluency task. Dement. Geriatr. Cogn. Disord. 45, 198–209. 10.1159/000487852 29886493

[B17] Koren R. Kofman O. Berger A. (2005). Analysis of word clustering in verbal fluency of school-aged children. Arch. Clin. Neuropsychol. 20, 1087–1104. 10.1016/j.acn.2005.06.012 16125896

[B18] Krippendorff K. (2004). Content Analysis: An Introduction to Its Methodology. Thousand Oaks, CA: Sage.

[B19] Landauer T. K. Foltz P. W. Laham D. (1998). An introduction to latent semantic analysis. Discourse Process. 25, 259–284.

[B20] Lanting S. Haugrud N. Crossley M. (2009). The effect of age and sex on clustering and switching during speeded verbal fluency tasks. J. Int. Neuropsychol. Soc. 15, 196–204. 10.1017/S1355617709090237 19203431

[B21] Levy O. Goldberg Y. Ramat-Gan I. (2014). Linguistic regularities in sparse and explicit word representations, in Proceedings of the 18th Conference on Computational Natural Language Learning (Baltimore, MD), 171–180.

[B22] Lezak M. D. (2004). Neuropsychological Assessment. Oxford: Oxford University Press.

[B23] Linz N. Tröger J. Alexandersson J. Konig A. (2017a). Using neural word embeddings in the analysis of the clinical semantic verbal fluency task, in IWCS 2017-12th International Conference on Computational Semantics (Montpellier), 1–7.

[B24] Linz N. Tröger J. Alexandersson J. Wolters M. König A. Robert P. (2017b). Predicting dementia screening and staging scores from semantic verbal fluency performance, in 2017 IEEE International Conference on Data Mining Workshops (New Orleans, LA), 719–728.

[B25] Locascio J. J. Growdon J. H. Corkin S. (1995). Cognitive test performance in detecting, staging, and tracking Alzheimer's disease. Arch. Neurol. 52, 1087–1099. 7487561 10.1001/archneur.1995.00540350081020

[B26] March G. E. Pattison P. (2006). Semantic verbal fluency in Alzheimer's disease: approaches beyond the traditional scoring system. J. Clin. Exp. Neuropsychol. 28, 549–566. 10.1080/13803390590949502 16624783

[B27] Maseda A. Lodeiro-Fernández L. Lorenzo-López L. Núñez-Naveira L. Balo A. Millán-Calenti J. C. (2014). Verbal fluency, naming and verbal comprehension: three aspects of language as predictors of cognitive impairment. Aging Mental Health. 18, 1037–1045. 10.1080/13607863.2014.908457 24797556

[B28] Mathuranath P. S. George A. Cherian P. J. Alexander A. Sarma S. G. Sarma P. S. (2003). Effects of age, education and gender on verbal fluency. J. Clin. Exp. Neuropsychol. 25, 1057–1064. 10.1076/jcen.25.8.1057.16736 14566579

[B29] Mayr U. Kliegl R. (2000). Complex semantic processing in old age: does it stay or does it go? Psychol. Aging. 15, 29–43. 10.1037/0882-7974.15.1.29 10755287

[B30] McDowd J. Hoffman L. Rozek E. Lyons K. E. Pahwa R. Burns J.. (2011). Understanding verbal fluency in healthy aging, Alzheimer's disease, and Parkinson's disease. Neuropsychology. 25, 210–225. 10.1037/a0021531 21381827

[B31] Methqal I. Marsolais Y. Wilson M. A. Monchi O. Joanette Y. (2019). More expertise for a better perspective: Task and strategy-driven adaptive neurofunctional reorganization for word production in high-performing older adults. Aging Neuropsychol. Cogn. 26, 190–221. 10.1080/13825585.2017.1423021 29334837

[B32] Mikolov T. Chen K. Corrado G. Dean J. (2013a). Efficient estimation of word representations in vector space, in Proceedings of Workshop at International Conference on Learning Representations (Scottsdale, AZ).

[B33] Mikolov T. Sutskever I. Chen K. Corrado G. S. Dean J. (2013b). Distributed representations of words and phrases and their compositionality, in Advances in Neural Information Processing Systems (Lake Tahoe, NV), 3111–3119.

[B34] Mikolov T. Yih W.-T. Zweig G. (2013c). Linguistic regularities in continuous space word representations, in Proceedings of the 2013 Conference of the North American Chapter of the Association for Computational Linguistics: Human Language Technologies (Atlanta, GA), 746–751.

[B35] Mioshi E. Dawson K. Mitchell J. Arnold R. Hodges J. R. (2006). The Addenbrooke's Cognitive Examination Revised (ACE-R): a brief cognitive test battery for dementia screening. Int. J. Geriatr. Psychiatry. 21, 1078–1085. 10.1002/gps.1610 16977673

[B36] Murphy K. J. Rich J. B. Troyer A. K. (2006). Verbal fluency patterns in amnestic mild cognitive impairment are characteristic of Alzheimer's type dementia. J. Int. Neuropsychol. Soc. 12, 570–574. 10.1017/s1355617706060590 16981610

[B37] Nicodemus K. K. Elvevåg B. Foltz P. W. Rosenstein M. Diaz-Asper C. Weinberger D. R. (2014). Category fluency, latent semantic analysis and schizophrenia: a candidate gene approach. Cortex. 55, 182–191. 10.1016/j.cortex.2013.12.004 24447899 PMC4039573

[B38] Pakhomov S. V. Eberly L. Knopman D. (2016). Characterizing cognitive performance in a large longitudinal study of aging with computerized semantic indices of verbal fluency. Neuropsychologia. 89, 42–56. 10.1016/j.neuropsychologia.2016.05.031 27245645 PMC4996679

[B39] Pakhomov S. V. Hemmy L. S. (2013). A computational linguistic measure of clustering behavior on semantic verbal fluency task predicts risk of future dementia in the Nun Study. Cortex. 55, 97–106. 10.1016/j.cortex.2013.05.009 23845236 PMC4402214

[B40] Pakhomov S. V. S. Hemmy L. S. Lim K. O. (2012). Automated semantic indices related to cognitive function and rate of cognitive decline. Neuropsychologia. 50, 2165–2175. 10.1016/j.neuropsychologia.2012.05.016 22659109 PMC3404821

[B41] Pakhomov S. V. S. Jones D. T. Knopman D. S. (2014). Language networks associated with computerized semantic indices. Neuroimage. 104, 125–137. 10.1016/j.neuroimage.2014.10.008 25315785 PMC4402216

[B42] Paula F. Wilkens R. Idiart M. Villavicencio A. (2018). Similarity measures for the detection of clinical conditions with verbal fluency tasks, in Proceedings of the 2018 Conference of the North American Chapter of the Association for Computational Linguistics: Human Language Technologies, Volume 2 (Short Papers) (New Orleans, LA), 231–235.

[B43] Prescott T. Newton L. D. Mir N. U. Woodruff P. W. R. Parks R. (2006). A new dissimilarity measure for finding semantic structure in category fluency data with implications for understanding memory organization in schizophrenia. Neuropsychology. 20, 685–699. 10.1037/0894-4105.20.6.685 17100513

[B44] Rohrer D. Wixted J. T. Salmon D. P. Butters N. (1995). Retrieval from semantic memory and its implications for Alzheimer's disease. J. Exp. Psychol. Learn. Mem. Cogn. 21, 1127–1139. 8744958 10.1037//0278-7393.21.5.1127

[B45] Sa S. Y. Chey J. Y. Suk J. S. (2011). Semantic structure of the elderly Koreans as assessed by category fluency test: effects of literacy and education. Korean J. Psychol. Gen. 30, 227–242. Retrieved from: https://www.dbpia.co.kr/

[B46] Salmon D. P. Thomas R. Pay M. Booth A. Hofstetter C. Thal L.. (2002). Alzheimer's disease can be accurately diagnosed in very mildly impaired individuals. Neurology. 59, 1022–1028. 10.1212/wnl.59.7.1022 12370456

[B47] Spreen O. Strauss E. (1998). A Compendium of Neuropsychological Tests: Administration, Norms, and Commentary. Oxford: Oxford University Press.

[B48] Sumiyoshi C. Ertugrul A. Anil Yagcioglu A. E. Sumiyoshi T. (2009). Semantic memory deficits based on category fluency performance in schizophrenia: similar impairment patterns of semantic organization across Turkish and Japanese patients. Psychiatry Research, 167(1-2):47–57. 19362375 10.1016/j.psychres.2007.12.009

[B49] Sung K. Gordon B. Vannorsdall T. D. Ledoux K. Pickett E. J. Pearlson G. D.. (2012). Semantic clustering of category fluency in schizophrenia examined with singular value decomposition. J. Int. Neuropsychol. Soc. 18, 565–575. 10.1017/S1355617712000136 22390863 PMC12922700

[B50] Tombaugh T. N. Kozak J. Rees L. (1999). Normative data stratified by age and education for two measures of verbal fluency: Fas and animal naming. Arch. Clin. Neuropsychol. 14, 167–177. 14590600

[B51] Tröster A. I. Fields J. A. Testa J. A. Paul R. H. Blanco C. R. Hames K. A.. (1998). Cortical and subcortical influences on clustering and switching in the performance of verbal fluency tasks. Neuropsychologia. 36, 295–304. 9665640 10.1016/s0028-3932(97)00153-x

[B52] Troyer A. K. (2000). Normative data for clustering and switching on verbal fluency tasks. J. Clin. Exp. Neuropsychol. 22, 370–378. 10.1076/1380-3395(200006)22:3;1-V;FT370 10855044

[B53] Troyer A. K. Moscovitch M. Winocur G. (1997). Clustering and switching as two components of verbal fluency: evidence from younger and older healthy adults. Neuropsychology. 11, 138–146. 9055277 10.1037//0894-4105.11.1.138

[B54] Troyer A. K. Moscovitch M. Winocur G. Leach L. Freedman M. (1998). Clustering and switching on verbal fluency tests in Alzheimer's and Parkinson's disease. J. Int. Neuropsychol. Soc. 4, 137–143. 9529823 10.1017/s1355617798001374

[B55] Voorspoels W. Storms G. Longenecker J. Verheyen S. Weinberger D. R. Elvevåg B. (2014). Deriving semantic structure from category fluency: clustering techniques and their pitfalls. Cortex. 55, 130–147. 10.1016/j.cortex.2013.09.006 24275165 PMC3983181

[B56] Weintraub S. Salmon D. Mercaldo N. Ferris S. Graff-Radford N. R. Chui H. (2009). The Alzheimer's disease centers' uniform data set (UDS): The neuropsychological test battery. Alzheimer Dis. Assoc. Disord. 23, 91–101. 10.1097/WAD.0b013e318191c7dd 19474567 PMC2743984

[B57] Wolters M. Kim N. Kim J.-H. MacPherson S. E. Park J. C. (2016). Prosodic and linguistic analysis of semantic fluency data: a window into speech production and cognition, in INTERSPEECH (San Francisco, CA), 2085–2089.

[B58] Wolters M. K. Johnson C. Campbell P. E. DePlacido C. G. McKinstry B. (2014). Can older people remember medication reminders presented using synthetic speech? J. Am. Med. Inform. Assoc. 22, 35–42. 10.1136/amiajnl-2014-002820 25080534 PMC4433370

[B59] Wu Z. Palmer M. (1994). Verbs semantics and lexical selection, in Proceedings of the 32nd Annual Meeting on Association for Computational Linguistics (Las Cruces, NM), 133–138.

